# Acquired hemophilia A and von Willebrand syndrome in a patient with late-onset systemic lupus erythematosus

**DOI:** 10.1186/2162-3619-3-21

**Published:** 2014-08-20

**Authors:** Christina Dicke, Katharina Holstein, Sonja Schneppenheim, Rita Dittmer, Reinhard Schneppenheim, Carsten Bokemeyer, Christof Iking-Konert, Ulrich Budde, Florian Langer

**Affiliations:** 1 II. Medizinische Klinik und Poliklinik, Universitätsklinikum Eppendorf, Martinistr. 52, 20246 Hamburg, Germany; 2 Gerinnungslabor, MEDILYS Laborgesellschaft mbH, c/o Asklepios Klinik Altona, Paul-Ehrlich-Str. 1, 22763 Hamburg, Germany; 3 Klinik und Poliklinik für Pädiatrische Hämatologie und Onkologie, Universitätsklinikum Eppendorf, Martinistr. 52, 20246 Hamburg, Germany; 4 III. Medizinische Klinik und Poliklinik, Universitätsklinikum Eppendorf, Martinistr. 52, 20246 Hamburg, Germany

**Keywords:** Acquired von Willebrand syndrome, Acquired hemophilia A, Systemic lupus erythematosus, Ultralarge von Willebrand factor plasma multimers

## Abstract

Acquired hemophilia A (AHA) and acquired von Willebrand Syndrome (AVWS) are both rare bleeding disorders that can be associated with lymphoproliferative or autoimmune diseases. AHA is uniformly caused by inhibitory autoantibodies against coagulation factor VIII (FVIII), while the pathophysiology of AVWS comprises several distinct mechanisms, including reduced synthesis, accelerated clearance, or increased proteolysis. In this regard, autoantibodies to von Willebrand factor (VWF) have been described in patients with systemic lupus erythematosus (SLE) or monoclonal gammopathy. Here, we report the case of a 71-year-old patient with a recent onset of spontaneous mucocutaneous and soft-tissue bleeding due to severely decreased FVIII and VWF. While there was no evidence for monoclonal gammopathy, specific IgG antibodies against both FVIII and VWF were detected. Furthermore, VWF multimer analysis revealed the presence of ultralarge plasma multimers and absence of the typical multimeric triplet structure, a finding consistent with decreased proteolytic processing of massively released, but rapidly cleared VWF. Both FVIII and VWF readily responded to immunosuppressive therapy with prednisolone. Interestingly, clinical and laboratory findings established the diagnosis of “late-onset SLE” in our patient. Thus, about 45 years after the first description of AVWS in a 12-year-old boy with SLE, we present another unusual case of concomitant autoimmune-mediated AHA and AVWS in an elderly SLE patient, which, to the best of our knowledge, has not been reported so far.

## Background

Acquired hemophilia A (AHA) and acquired von Willebrand syndrome (AVWS) are rare bleeding disorders. They both affect mostly elderly people and are associated with significant morbidity and mortality. While AHA is uniformly caused by inhibitory autoantibodies against coagulation factor VIII (FVIII), the pathophysiology of AVWS comprises several distinct mechanisms. These include, but are not limited to, reduced synthesis, accelerated clearance due to circulating autoantibodies or adsorption onto tumor cells or platelets, and increased shear-dependent cleavage by ADAMTS13 or by other more non-specific proteases such as plasmin. AVWS may thus result from quantitative and/or qualitative abnormalities of plasma VWF [1].

Malignancy and autoimmune diseases are among the most frequently reported underlying conditions in patients with AHA, but more than half of the cases remain idiopathic. In contrast, virtually all cases of AVWS are secondary to another disease, including hematological, lymphoproliferative, cardiovascular, and autoimmune disorders as well as hypothyroidism [2-4]. In this regard, it is noteworthy that the first description of AVWS has been in a boy suffering from systemic lupus erythematosus (SLE) [5] with subsequent studies clearly implicating the production of VWF autoantibodies in the pathogenesis of SLE-associated AVWS [6-11].

Although VWF has specific FVIII binding sites and functions as a protective carrier protein for FVIII within the circulation, which may at least theoretically serve as the molecular basis for a common autoimmune reaction against the VWF/FVIII complex, a case of concomitant immune-mediated AHA and AVWS has not been previously reported.

## Case presentation

**Figure 1:**
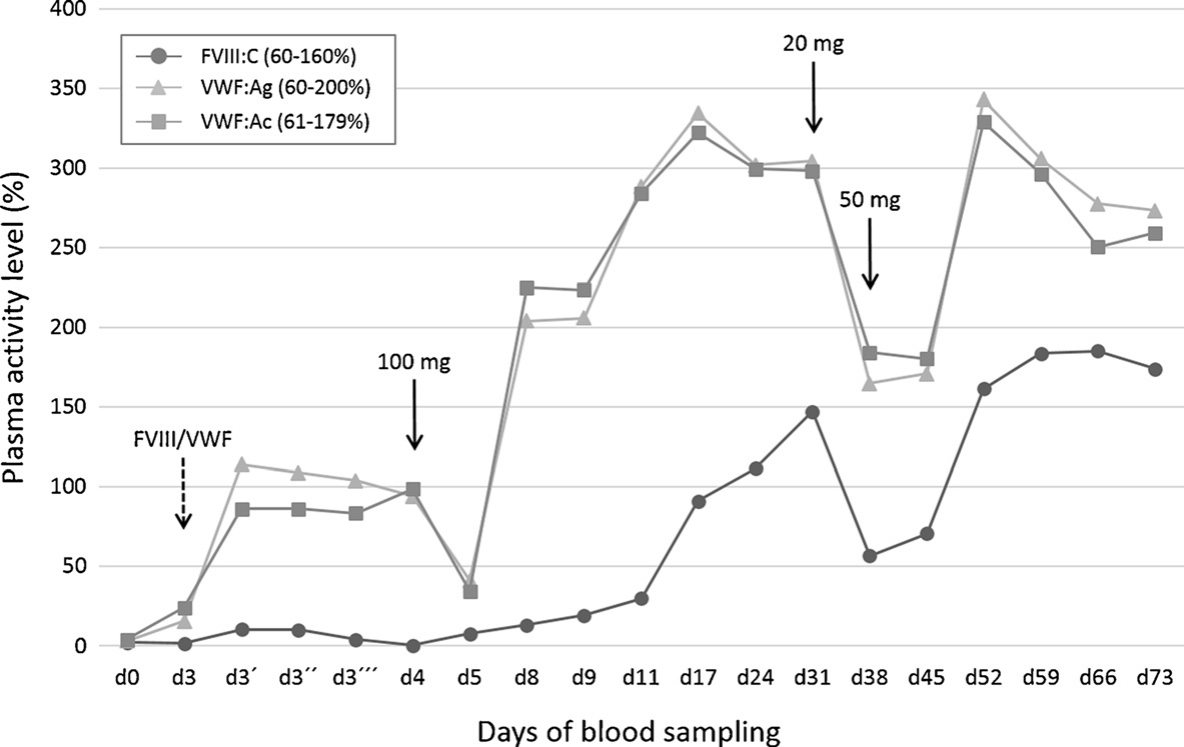
**Plasma levels of factor VIII clotting activity (FVIII:C) and von Willebrand factor parameters (VWF:Ag and VWF:Ac) at presentation and during course of treatment.** The first set of data points (d0) shows FVIII and VWF levels at initial presentation, while the second set of data points (d3) shows FVIII and VWF levels immediately before the administration of 2,000 IU of FVIII/VWF concentrate, as indicated by the dashed arrow. To assess incremental recovery and residence time of the infused FVIII/VWF, subsequent blood samples were drawn 15 min (d3´), 60 min (d3´´), and 120 min (d3´´´) after concentrate administration. One day later (d4), immunosuppressive therapy with prednisolone was started at a daily dose of 100 mg, which was gradually tapered to 20 mg on d31, as indicated by the solid arrows. One week later, there was a steep decline in FVIII and VWF, both of which readily responded to re-escalating the prednisolone dose to 50 mg per day.

A 71-year-old man was admitted to our hospital due to recent onset of diffuse gum bleeding. Macroscopic hematuria and spontaneous bruises had also occurred one month prior to the current presentation. A minor trauma during physical exercise had led to an extensive hemarthrosis of the left elbow. Previously, the patient had also complained about recurrent skin rashes, myalgia, arthralgia, and fatigue. His prior medical history included arteriosclerosis, paresthesia of both legs of unknown etiology, and asbestosis with no evidence for pleural mesothelioma. His family history was negative for a bleeding diathesis, and he did not take any medication.On admission (day 0), coagulation tests revealed a significantly prolonged activated partial thromboplastin time (APTT) of 79 sec (normal range, 25-38 sec) with no correction upon 1:1 (vol:vol) mixing of the patient with normal human plasma. FVIII clotting activity (FVIII:C) was reduced to 2% (normal range, 60-160%) [Figure 1]. A Nijmegen-modified Bethesda and an enzyme-linked immunosorbent assay (ELISA) revealed a FVIII inhibitor of 4.6 Bethesda units (BU) (normal range, <0.5 BU) and 1.37 optical densities (OD) (normal plasma pool, 0.24 OD), respectively. These findings clearly established the diagnosis of AHA.

However, further laboratory work-up revealed maximally prolonged closure times of >300 sec in the PFA-100® (platelet function analyzer, Dade-Behring) using both the collagen/epinephrine (normal range, 84-160 sec) and the collagen/ADP cartridge (normal range, 68-121 sec), which suggested severely impaired shear-dependent primary hemostasis. In addition, platelet agglutination in the patient’s platelet-rich plasma induced by 1.2 or 1.5 mg/mL ristocetin was dramatically reduced to 4% and 24%, respectively (normal ranges, 60-95% and 75-100%, respectively), further indicating a specific defect in plasma VWF. Consistently, VWF antigen (VWF:Ag) was <3% (normal range, 60-200%), and VWF activity (VWF:Ac), as determined by the INNOVANCE® GPIb binding assay (Siemens Healthcare), was 4% (normal range, 61-179%) [Figure 1]. The ratio of the prothrombin time (INR, 0.93) and the platelet count (206 × 10^3^/μL) were normal.

**Figure 2:**
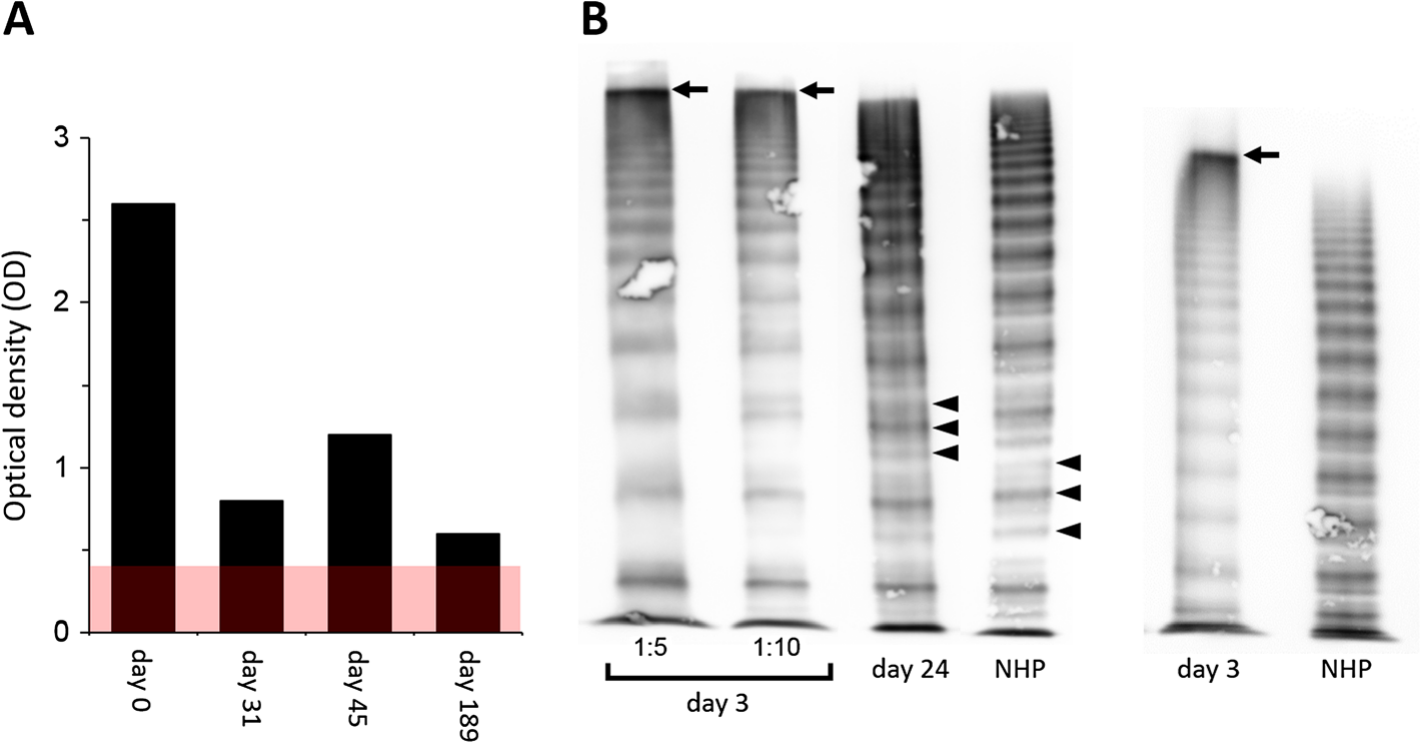
**Results of the in-house ELISA for the detection of anti-VWF-IgG and multimer analysis. (A)** Immobilized recombinant human VWF derived from CHO cells was used to measure IgG autoantibodies to VWF. The titer was 2.6 OD at presentation (day 0) and decreased to 0.8 OD during effective prednisolone therapy (day 31). A steep decline in VWF on day 38 following reduction of the daily prednisolone dose to 20 mg a week earlier was accompanied by a temporary increase in the anti-VWF-IgG titer to 1.2 OD on day 45. After six months of follow-up, the titer had decreased to 0.6 OD, but was still above the reference range of the ELISA as indicated by the shaded red area. **(B)** Before the initiation of prednisolone, presence of unusually large VWF plasma multimers (arrows) and absence of typical VWF triplets (arrow heads) indicated decreased proteolysis of newly released, but rapidly cleared VWF (day 3). The multimeric pattern normalized during effective immunosuppressive therapy with prednisolone (day 24). A high-resolution 1.6% agarose gel (left four lanes) and a low-resolution 1.2% agarose gel (right two lanes), which allows for better migration of the larger multimers into the gel, are shown. Different dilutions of the patient plasma obtained on day 3 are indicated in the high-resolution gel. NHP denotes normal human plasma.

Because severely decreased VWF parameters could not be explained by the low-titer FVIII inhibitor, we used a novel in-house ELISA to explore the concomitant presence of a VWF autoantibody [12]. Briefly, recombinant human VWF (rhVWF) purified from culture supernatants of transfected Chinese hamster ovary (CHO) cells was bound to high binding ELISA plates and left in the refrigerator until use. Following incubation of diluted plasma samples (1:50 in phosphate-buffered saline/albumin) for 90 min at 37°C, plates were washed and incubated with a peroxidase-labeled anti-human IgG antibody and developed with o-phenylenediamine dihydrochloride. The reference was a 1:50 diluted normal plasma pool. The cut off was defined as 2 x the OD of this plasma pool.At presentation (day 0), we detected an anti-VWF IgG antibody of 2.6 OD (normal range, <0.4 OD) [Figure 2A]. Importantly, addition of rhVWF to the patient plasma completely blocked this reaction [data not shown], further confirming specificity of the ELISA. Multimer analysis was carried out on a plasma sample obtained on day 3, on which VWF parameters had spontaneously increased to 15-25% [Figure 1], revealing the presence of ultralarge VWF plasma multimers and absence of the typical multimeric triplet structure [Figure 2B].The patient received 2,000 IU of a VWF-containing FVIII concentrate (Haemate® P, CSL Behring) on day 3. While there was only a marginal and short-lasting increment in FVIII:C, VWF:Ag and VWF:Ac increased to 114% and 86%, respectively, and temporarily steadied at these levels for 24 hrs before declining back to 34-41% on day 5 [Figure 1].

Further diagnostic work-up revealed Coombs-positive hemolytic anemia (hemoglobin, 12.3 g/dL) and high-titer antinuclear antibodies (ANAs) at >1:5,120 (normal range, <1:80) with a fine-speckled staining pattern. Anti-double-stranded DNA antibodies and antibodies to extractable nuclear antigens were negative, while IgG antibodies to cardiolipin and β2-glycoprotein-I were significantly increased to 256 U/mL and 564 U/mL, respectively (normal ranges, <10 U/mL). Furthermore, the patient had a mild decrease in complement C3 of 79 mg/dL (normal range, 90-180 mg/dL) and borderline lymphocytopenia of 21.7% (normal range, 20-45%). A dilute Russell’s viper venom time for the detection of lupus anticoagulant was 38 sec (normal range, 30-40 sec), while the results of an APTT-based assay were inconclusive, probably due to the FVIII inhibitor [13].

Computed tomography of the chest and abdomen excluded an underlying malignancy. Thus the diagnosis of “late-onset SLE” was made based on the following clinical and laboratory criteria [14]: skin rash, Coombs-positive hemolytic anemia, positive antiphospholipid antibodies and ANAs, lymphocytopenia, and C3 decrease.Immunosuppressive therapy with prednisolone was initiated on day 4 at a daily dose of 100 mg, which resulted in rapid elevations of VWF:Ag and VWF:Ac within four days and a more delayed recovery of FVIII:C to normal levels within 13 days into treatment [Figure 1]. Normalization of VWF parameters was accompanied by the loss of ultralarge plasma multimers and appearance of typical VWF triplets on day 24 [Figure 2B]. Moreover, the anti-FVIII- and anti-VWF-IgG titers decreased to 0.21 OD and 0.8 OD, respectively, on day 31.The daily prednisolone dose was gradually tapered and reduced to 20 mg on day 31, which resulted in a sharp decline of VWF:Ag, VWF:Ac, and FVIII:C one week later [Figure 1] and was accompanied by a temporary increase in the anti-FVIII- and anti-VWF-IgG titers to 0.63 OD and 1.15 OD, respectively, on day 45. All parameters rapidly responded to re-escalating the daily prednisolone dose to 50 mg on day 38.

Azathioprine and hydroxychloroquine were added at daily doses of 50 mg and 200 mg, respectively, to spare corticosteroids and maintain remission. However, azathioprine had to be discontinued after one month due to suspected drug fever. In addition, hydroxychloroquine was discontinued ten weeks later based on the patient’s preference and more non-specific side effects such as myalgia, dizziness, and general discomfort. Nevertheless, the prednisolone dose could be gradually tapered to a maintenance dose of 5 mg per day. At the latest follow-up, exactly one year after the initial presentation, the patient was still in complete remission with regard to his acquired bleeding disorders.

## Discussion

Here, we describe the case of an elderly patient with acquired inhibitors against both FVIII and VWF. To the best of our knowledge, a similar case has not been previously reported. Intriguingly, clinical and laboratory work-up revealed “late-onset SLE” as the most probable underlying condition. Development of this autoimmune disorder in a 71-year-old man in itself represents a quite unusual observation.

Typical clinical symptoms of AHA include spontaneous (muco)cutaneous and soft-tissue bleeding such as observed in our patient. AHA is characterized by a prolonged APTT due to isolated FVIII deficiency that cannot be corrected by mixing with normal human plasma. The diagnosis is confirmed by the detection of a FVIII inhibitor by either Bethesda assay or ELISA [15]. Our patient, however, not only presented with severely decreased FVIII:C, but also with dramatically reduced plasma VWF. In fact, based on the constellation of these parameters, we initially suspected AVWS due to monoclonal gammopathy as the most likely diagnosis. In this hemostatic disorder, the primary pathophysiological event is rapid clearance of plasma VWF by a yet not completely understood mechanism, while the decrease in circulating FVIII is secondary to depletion of its carrier protein, VWF [3]. Consequently, FVIII:C is usually responsive to *ex vivo* mixing with normal human plasma. Interestingly, our in-house ELISA did not detect anti-VWF-IgG in 14 patients with AVWS due to IgG monoclonal gammopathy of unknown significance (MGUS) [12], suggesting that the paraprotein itself does not (always) function as a circulating VWF inhibitor. In our patient, the severely decreased FVIII:C in the presence of only moderately decreased VWF levels on day 3 may also be considered unusual for MGUS-associated AVWS. Finally, a monoclonal paraprotein was ruled out by serum immunofixation in our patient.Using a modified Bethesda assay, we could not detect any functional interference of the IgG autoantibody with VWF binding to immobilized collagen [data not shown]. A shortened half-life with accelerated clearance of the antibody-opsonized VWF by the reticuloendothelial system was thus the most plausible mechanism of VWF depletion in our patient. The findings of multimer analysis may be supportive of this hypothesis, because the presence of ultralarge plasma multimers and the absence of typical triplets on day 3 are consistent with decreased ADAMTS13-mediated proteolysis of massively released, but rapidly cleared VWF [Figure 2B]. In this regard, however, the effect of FVIII/VWF substitution on day 3 warrants closer attention.

The plasma-derived FVIII/VWF concentrate (Haemate® P) was dosed according to its FVIII:C content. Consequently, the patient received 2,000 IU of FVIII:C and approximately 4,800 IU of VWF:RCo, the latter of which corresponded to a body weight-adjusted dose of 60-65 IU/kg. Assuming an increase in plasma VWF of 1-2% per each IU infused per kg of body weight in patients with congenital von Willebrand disease, the recovery observed on day 3 appears adequate. Furthermore, the subsequent decline in VWF parameters is consistent with a half-life of up to 24 hrs. In fact, VWF parameters appeared to stabilize for almost a day at 100% before declining back to <50% two days after the administration of FVIII/VWF concentrate. These findings clearly suggest that the patient’s IgG autoantibody accelerated clearance of self-produced VWF, while it did not affect the purified plasma-derived VWF present in Haemate® P.

FVIII:C showed only a marginal response to FVIII/VWF substitution, a finding characteristic for AHA and further supporting our conclusion that the patient had two distinct immune responses, one against FVIII and one against VWF. Consistently, following initiation of prednisolone therapy on day 4, VWF parameters normalized within four days, while FVIII:C showed a more delayed response with normal values not reached before almost two weeks into treatment.

So far, only 16 cases of AVWS related to SLE have been reported [3,10,11,16]. In these patients, different patterns of VWF plasma multimers have been observed. While loss of larger plasma multimers was documented in six patients, corresponding to a type-2 pattern [3,10], multimers were completely absent in two patients [3], corresponding to a type-3 pattern. In our patient, first multimer analysis was carried out on day 3, on which VWF parameters had spontaneously increased from <5% to 15-25%. While a type-2 pattern could be ruled out at first sight, the presence of a condensed band at exceedingly high molecular weight suggested the presence of ultralarge VWF plasma multimers. This finding was confirmed using a low-resolution agarose gel [Figure 2B]. Occurrence of ultralarge VWF plasma multimers has been described in patients with thrombotic-thrombocytopenic purpura (TTP) in whom endothelium-derived VWF is not processed due to an inherited or acquired deficiency of the VWF-cleaving metalloproteinase, ADAMTS13 [17]. Consistent with a similar mechanism of decreased ADAMTS13-mediated VWF cleavage in our patient, proteolytic subbands accounting for the typical multimeric triplet structure were absent at presentation, but appeared following remission induction. We hypothesize that rapid clearance of the newly released VWF by the IgG autoantibody prevented its proper proteolytic processing. Massive release of VWF, which is likely to occur in SLE patients due to an acute-on-chronic inflammation of the vascularized connective tissue [18], may have temporarily overruled the clearing kinetics of the IgG autoantibody, allowing for visualization of the slightly increased VWF on day 3. Additionally (or alternatively), binding of the autoantibody to VWF may have conferred steric hindrance, thereby inhibiting proper enzyme-substrate interactions. In this regard, the condensed high-molecular-weight band may be composed of, or at least contain, IgG-VWF immune complexes. However, normal migration of the low and intermediate sized plasma multimers may argue against this hypothesis. Nevertheless, studies addressing these issues are currently ongoing in our laboratory.

Previous studies on the presence of VWF autoantibodies using immunoassays with immobilized plasma-derived VWF have been prone to false-positive results due to cross-reactivity of plasma isoagglutinins with ABO blood group antigens present on VWF [19]. To improve specificity, we have developed an in-house ELISA with rhVWF derived from the transfected mammalian, but non-human CHO cell line. Interestingly, eleven out of 112 healthy blood donors (10%) were tested positive with ODs of 0.4-0.6. Since reactions could be blocked by exogenous addition of VWF, signals seemed to be specific for anti-VWF-IgG. As expected, there was no ABO blood group dependency of this reaction [12].

Considering that the patient had two severe and potentially life-threatening bleeding disorders, AHA and AVWS, the hemorrhagic phenotype may be judged as quite moderate. In this regard, it could be speculated that the concomitant presence of elevated antiphospholipid antibodies partially counterbalanced the patient’s bleeding diathesis through the promotion of a thrombogenic state [20,21].

## Conclusion

In summary, about 45 years after its first description in a 12-year-old boy with SLE, we describe another unusual case of AVWS in an elderly patient with “late-onset SLE”. Although FVIII specifically binds to VWF within the circulation, to our knowledge this is the first case report describing concomitant autoimmune-mediated AHA and AVWS in a single patient.

In patients with suspected AHA, laboratory assessment of circulating VWF (i.e. through quantification of its antigen and activity levels) may be regarded as common clinical practice to rule out AVWS as an alternative diagnosis. Based on our experience, however, the future diagnostic work-up of patients with AVWS and severely reduced plasma FVIII should also include specific immunologic (i.e. ELISA) or functional inhibitor tests (i.e. mixing study or Bethesda assay) to safely exclude concomitant AHA.

## Consent

Written informed consent was obtained from the patient for publication of this case report and any accompanying images. A copy of the written consent is available for review by the Editor-in-Chief of this journal.

## Abbreviations

AHA: Acquired hemophilia A; AVWS: Acquired von Willebrand syndrome; VWF: Von Willebrand factor; FVIII: Factor VIII; SLE: Systemic lupus erythematosus; APTT: Activated partial thromboplastin time; FVIII:C: Factor VIII clotting activity; ELISA: Enzyme-linked immunosorbent assay; BU: Bethesda units; OD: Optical densities; PFA: Platelet function analyzer; VWF:Ag: Von Willebrand factor antigen; VWF:Ac: Von Willebrand factor activity; rhVWF: Recombinant human von Willebrand factor; CHO: Chinese hamster ovary; ANA: Antinuclear antibodies; MGUS: Monoclonal gammopathy of unknown significance; TTP: Thrombotic-thrombocytopenic purpura.

## Competing interests

We declare having no conflict of interest. This case report has not been published elsewhere nor is it under consideration for publication elsewhere. Consent to publish patient related data has been obtained by the patient.

## Authors’ contributions

All authors have read and approved the final version of the manuscript. CD, KH, and FL cared for the patient, gathered and interpreted all relevant clinical and experimental data, and wrote the manuscript. SS, RD, RS, and UB developed the anti-VWF-IgG in-house ELISA, carried out and interpreted all laboratory studies relevant to the diagnosis of AHA and AVWS, including multimer analysis, and critically revised the manuscript. CB and CIK oversaw the clinical management of the patient, interpreted findings, and critically revised the manuscript.

## References

[B1] Tiede A Rand JH Budde U Ganser A Federici AB How I treat the acquired von Willebrand syndrome Blood 2011 117 6777 6785 10.1182/blood-2010-11-297580 21540459

[B2] Franchini M Mannucci PM Acquired haemophilia A: a 2013 update Thromb Haemost 2013 110 1114 1120 10.1160/TH13-05-0363 24008306

[B3] Michiels JJ Berneman Z Gadisseur A van der Planken M Schroyens W Budde U van Vliet HH Immune-mediated etiology of acquired von Willebrand syndrome in systemic lupus erythematosus and in benign monoclonal gammopathy: therapeutic implications Semin Thromb Hemost 2006 32 577 588 10.1055/s-2006-949663 16977568

[B4] Federici AB Budde U Castaman G Rand JH Tiede A Current diagnostic and therapeutic approaches to patients with acquired von Willebrand syndrome: a 2013 update Semin Thromb Hemost 2013 39 191 201 10.1055/s-0033-1334867 23397553

[B5] Simone JV Cornet JA Abildgaard CF Acquired von Willebrand’s syndrome in systemic lupus erythematosus Blood 1968 31 806 812 4172730

[B6] Gazengel C Prieur AM Jacques C Buriot D Nedellec J Josso F Antibody-induced von Willebrand syndrome: inhibition of VIII VWF and VIII AGN with sparing of VIII AHF by the autoantibody Am J Hematol 1978 5 355 363 10.1002/ajh.2830050411 313704

[B7] Soff GA Green D Autoantibody to von Willebrand factor in systemic lupus erythematosus J Lab Clin Med 1993 121 424 430 8445290

[B8] Viallard JF Pellegrin JL Vergnes C Borel-Derlon A Clofent-Sanchez G Nurden AT Leng B Nurden P Three cases of acquired von Willebrand disease associated with systemic lupus erythematosus Br J Haematol 1999 105 532 537 10.1111/j.1365-2141.1999.01360.x 10233433

[B9] Casonato A Pontara E Doria A Bertomoro A Cattini MG Gambari PF Girolami A Lack of multimer organization of von Willebrand factor in an acquired von Willebrand syndrome Br J Haematol 2002 116 899 904 10.1046/j.0007-1048.2002.03341.x 11886398

[B10] Hong S Lee J Chi H Lee C Nah S Kim Y Oh J Moon H Yoo B Systemic lupus erythematosus complicated by acquired von Willebrand’s syndrome Lupus 2008 17 846 848 10.1177/0961203308089429 18755868

[B11] Kasatkar P Ghosh K Shetty S An atypical manifestation of acquired von Willebrand syndrome (AVWS) associated with systemic lupus erythematosus (SLE) Ann Hematol 2014 93 173 175 10.1007/s00277-013-1767-4 23625134

[B12] Budde U Rausch T El-Abd Müller H Langer F Obser T Schneppenheim S Ditter R Schneppenheim R Development of a new ELISA test for the detection of auf auto- and alloantibodies in patients with von Willebrand disease Poster presented at *58*^*th*^* Annual Meeting of the Society of Thrombosis and Haemostasis Research.* Vienna, Austria; 2014. http://www.gth-online.org/home/jahrestagungen/gth-tagung-2014.php

[B13] Tripodi A Testing for lupus anticoagulants: all that a clinician should know Lupus 2009 18 291 298 10.1177/0961203308101436 19276296

[B14] Hochberg MC Updating the American College of Rheumatology revised criteria for the classification of systemic lupus erythematosus Arthritis Rheum 1997 40 1725 9324032 10.1002/art.1780400928

[B15] Sborov DW Rodgers GM How I manage patients with acquired haemophilia A Br J Haematol 2013 161 157 165 10.1111/bjh.12228 23373521

[B16] Jimenez AR Vallejo ES Cruz MZ Cruz AC Miramontes JV Jara BS Rituximab effectiveness in a patient with juvenile systemic lupus erythematosus complicated with acquired Von Willebrand syndrome Lupus 2013 22 1514 1517 10.1177/0961203313502862 23989733

[B17] Huck V Schneider MF Gorzelanny C Schneider SW The various states of von Willebrand factor and their function in physiology and pathophysiology Thromb Haemost 2014 111 598 609 10.1160/TH13-09-0800 24573248

[B18] Lenting PJ Casari C Christophe OD Denis CV von Willebrand factor: the old, the new and the unknown J Thromb Haemost 2012 10 2428 2437 10.1111/jth.12008 23020315

[B19] Matsui T Fujimura Y Nishida S Titani K Human plasma alpha 2-macroglobulin and von Willebrand factor possess covalently linked ABO(H) blood group antigens in subjects with corresponding ABO phenotype Blood 1993 82 663 668 7687165

[B20] Ruffatti A Del Ross T Ciprian M Nuzzo M Rampudda M Bertero MT Bergia R Caramaschi P Biasi D Capsoni F Montaguti L Ruffini R Brucato A Picillo U Fanelli V Riccieri V Piccoli A Valesini G Doria A Meroni PL Tincani A Risk factors for a first thrombotic event in antiphospholipid antibody carriers. A multicentre, retrospective follow-up study Ann Rheum Dis 2009 68 397 399 10.1136/ard.2008.096669 18812393

[B21] Tektonidou MG Laskari K Panagiotakos DB Moutsopoulos HM Risk factors for thrombosis and primary thrombosis prevention in patients with systemic lupus erythematosus with or without antiphospholipid antibodies Arthritis Rheum 2009 61 29 36 19116963 10.1002/art.24232

